# Adapting to the emotional complexity of palliative care communication: Palliative care clinicians’ experiences

**DOI:** 10.1017/S1478951524001883

**Published:** 2025-01-21

**Authors:** Anna Niederhauser, Martina Egloff, Steffen Eychmueller, Sofia C. Zambrano

**Affiliations:** 1 Institute of Social and Preventive Medicine (ISPM), University of Bern, Bern, Switzerland; 2 University Center for Palliative Care, Inselspital, University Hospital Bern, University of Bern, Bern, Switzerland

**Keywords:** Health professionals, coping, communication, emotions, palliative care

## Abstract

**Objectives:**

Communication is essential to medical care and is key in improving patient outcomes. We aimed to understand clinicians’ emotions when communicating with patients in palliative care (PC) and the evolution of their communication skills.

**Methods:**

Between October and November 2021, 231 Swiss PC clinicians participated in an online cross-sectional survey (65% nurses, 35% physicians). Three questions invited participants to reflect on the development of their communication skills and on their emotions when communicating with patients in PC. Answers to these questions were analyzed thematically.

**Results:**

*Constantly adapting to complex emotions in communication* was the overarching theme. Three main themes further allowed for an understanding of different communication challenges: *emotions as a dynamic compass, not always under control*, and *evolving comfort and competence through personal and professional growth*. In *evolving comfort and competence*, 6 strategies helped clinicians overcome fears and improve their confidence in communication: learning by doing and formal training, integrating life experiences and gaining insight from private life, taking time, collaborating and connecting with peers, acknowledging individuality, and connecting with one’s own and patients’ emotions.

**Significance of results:**

Participants described powerful emotional experiences when communicating with patients in PC, underscoring the emotional labor involved in PC communication. Our study highlights the need to re-conceptualize emotions as a valuable resource rather than a hindrance in clinical practice. The interplay between personal and professional identities in shaping communication skills, emphasizes the importance of emotional competence as a core professional skill. As clinicians often develop these skills individually, our findings suggest a need for earlier integration of emotional competence training in medical education, along with ongoing support through collaboration, and improved allocation of time resources, to enhance clinician well-being and patient care.

## Introduction

Effective communication is essential to medical care and is known to be key in improving patient outcomes in palliative care (PC) (Parker et al. 2007). The field of health communication has long recognized the complexities of clinician-patient interactions, particularly in emotionally charged contexts (Ong et al. 1995; Street et al. 2009). Research in this area has highlighted the importance of patient-centered communication (Epstein and Street 2007), shared decision-making (Elwyn et al. 2012), and the impact of communication on patient outcomes (Street et al. 2009). In the context of PC, these general principles of effective communication take on added significance due to the emotional intensity and existential nature of end-of-life (EOL) discussions (Back et al. 2005; Tulsky 2005).

EOL communication poses challenges to different health professionals including nurses and physicians from different specialty fields (e.g. Alqahtani and Mitchell 2019; Billings et al. 2009; Fulmer et al. 2018; Granek et al. 2013; Moir et al. 2016; Oh et al. 2019; Pollock and Wilson 2015; Zhang et al. 2022). Among the different challenges are: time pressure, lack of education, patient-related factors, as well as uncertainties surrounding illness progression (Blackwood et al. 2019; Brighton and Bristowe 2016; Oh et al. 2019; Pollock and Wilson 2015). The emotional impact of working in PC, particularly in communicating within an area of suffering and uncertainty, also puts clinicians at risk of stress, and even burnout (Horn and Johnston 2020; Koh et al. 2015; Parola et al. 2017; Pereira et al. 2011). This risk is further compounded by increasing professional pressure due to the rising demand for PC (Etkind et al. 2017; Gesundheitspolitik 2020) and staff shortages (Lupu et al. 2018; Merçay and Grünig, 2016; Zhang et al. 2020).

Education is recognized as central to the development of effective communication skills (De Haes and Teunissen 2005), and different programs to improve communication skills have been developed and found to be successful in supporting confidence and improving the communication skills of PC clinicians (Clayton et al. 2012 Fuoto and Turner 2019; Goldsmith et al. 2013; Harnischfeger et al. 2022; Wittenberg-Lyles et al. 2014). Notable among these is the COMFORT model, which specifically addresses the emotional aspects of PC communication (Wittenberg-Lyles et al. 2014).

PC clinicians are considered to be among the most skilled communicators in health care due to communication being the cornerstone of PC (Wittenberg et al. 2015). However, understanding how PC clinicians acquire and refine their communication skills, the strategies that have supported their development, and how they cope with the emotional challenges inherent in their work remains a key area for research. Therefore, we aimed to explore PC clinicians’ experiences of EOL communication, focusing on how their comfort and communication skills have evolved over time and how they manage the challenges of their role. By examining these aspects, we can gain deeper insights into what helps clinicians excel in such a demanding yet crucial field, potentially informing future training and support strategies for healthcare professionals engaged in EOL care.

## Methodology

### Design

Swiss PC clinicians were invited to participate in an online cross-sectional survey about their comfort and competence with EOL communication. Among the items, open-ended questions invited participants to reflect on the development of their communication skills and on their emotional experiences when communicating with patients. This manuscript reports the qualitative analysis of responses to the open-ended questions. The Bernese Cantonal Ethical commission exempted the study from ethical review (Req-2020-00611).

### Data collection

All health professionals working in PC in Switzerland who were attending the Swiss National Palliative Care Congress in 2021 were invited to participate between October and November 2021. After approval from palliative.ch (Swiss Association of Palliative Care), attendees received an invitation to complete the survey via the conference organizing body, Medworld. In addition, palliative.ch sent the invitation to all physicians in the *physician group of interest* of the association. Two weeks after the initial email, a reminder was sent.

### Instrument

The survey was made available in German, French, and English via Survey Monkey. Prior to participation, participants read the study aims and a statement on anonymity and confidentiality. Responses to the following questions were analyzed: (1) “*How much has your level of comfort with end of life and PC conversations changed across your career? For example, which topics have become easier or more difficult to discuss across your career and why?*”, (2) “*What emotions have you experienced before, during, and after talking with patients in PC?*”, and (3) “*Are there any other comments about the topic of this survey, which you would like to share with us?*”. Following instructions for qualitative surveys (Braun et al. 2021; Terry and Braun 2017), participants were prompted to write as many details as possible and were given wide spaces to write their answers.

### Participants

The survey was completed by 231 nurses (65%) and physicians (35%) of which the majority were women (79%) and were 50 years old in average. Participants came from almost all Swiss cantons of which Zurich had the largest proportion (25%), and worked in different PC settings, with a majority working on specialist PC units (19.42%). The majority had training in specialist PC (nurses 81%, physicians 83%) and had PC experience of 12.4 years in average (Table 1).

**Table 1.** Participant characteristics (*n* = 231)

Variable	Participants	%
Gender, *n* (%)		
Female	182	78.8
Male	49	21.2
Language, *n* (%)		
German	190	82.3
French	37	16
English	4	1.7
Occupation, *n* (%)		
Medicine	81	35.1
Nursing	150	64.9
Work dedication, *n* (%)		
Full-time	151	65.4
Part-time	79	34.2

### Data analysis

The dataset was fully analyzed employing reflexive thematic analysis (Braun and Clarke 2022) by AN, a female resident physician, with support from SCZ, a psychologist experienced in PC and in qualitative research. In a first step, AN read all responses actively and repeatedly, initially per participant and afterward per question, to achieve familiarization. Notes were taken at this stage writing down initial patterns and meanings in the data. In a second step, all responses were imported into NVivo12 and coded following an inductive coding approach, systematically coding important data features through the entire dataset. Semantic (explicit contents) as well as latent codes (interpreting meanings in the data) were generated. Off-topic comments mainly from the last question or “thank you” messages were excluded from analyses. As AN was new to qualitative research, to ensure that the coding process was well understood, the first 10 responses per question were coded together by AN and SZ. Initial codes were discussed and when needed, further instructions on the coding process were considered. The third step involved organizing the codes into groups and discussing potential themes and subthemes across the dataset. The fourth step involved reviewing themes and linked data extracts with support of cognitive maps to ensure the data fit into the themes. The fifth step consisted of naming the themes and subthemes. Steps 3–5 were continuously discussed and adjusted between AN and SZ. The final step involved writing the results. We approached the analysis from a critical realist and contextualist stance, acknowledging that while there is a reality independent of our perceptions, our understanding of it is influenced by our interpretations and social contexts.

## Results

In our analysis of PC clinicians’ experiences with EOL communication, we identified a complex emotional context that evolved over time. As the overarching theme, we identified “constantly adapting to complex emotions in communication,” reflecting how clinicians experienced a variety of emotions and how they actively engaged in understanding, handling, and responding to them. Within this overarching theme, we identified 3 main themes: (1) Emotions as a dynamic compass, (2) Not always under control, and (3) Evolving comfort and competence through personal and professional growth.

### Constantly adapting to complex emotions in communication

Participants experienced the entire palette of emotions when communicating with patients in PC. This emotional complexity seemed ever present, and the contrasting nature of participants’ emotional experiences required continuous adaptation not only between patients but at times within the same consultation. While persisting and demanding, most clinicians described having developed different skills to manage this emotional complexity effectively.

*“The different human feelings: love, hate, empathy, security, stress, joy, pleasure, sadness, anger, disgust, fear, helplessness, dissatisfaction, pride, blocked, shame, guilt, peace …. Depending on the conversations and self-involvement, work stress, etc., you can feel any different feelings.”* (Physician, 20 years of PC exp, male) P150

*“Deep gratitude – for many thoughts and feelings I have heard, but also for the time spent with the patients and their relatives – I learn a lot through them.”* (Nurse, 22 years of PC exp, female) P212

*“A feeling of being supportive in the process, a confidant, a person who gives support and strength, and who is a competent contact person.”* (Nurse, 15 years of PC exp, male) P279

*“Will I be able to meet the needs, will I be able to respond? A feeling of loneliness in view of the magnitude of the problems. A greater difficulty to breathe, to distinguish between work and home.”* (Nurse, 11 years of PC exp, female) P220

### Theme 1. Emotions as a dynamic compass

Participants experienced a range of emotions throughout patient interactions, often employing them as a dynamic compass, guiding their interactions and helping them shape their responses. Clinicians shared how emotions helped them develop deep connections and to empathize with their patients, which they saw as enhancing the quality of care but which also increased their own vulnerability. Specific emotions appeared in noticeable patterns:

Before conversations, clinicians often felt uncertain and tense, emphasizing the need for preparation and focus.

*“The feelings before the conversation depend on any previous contacts/conversations. Feelings can accordingly be very pleasant, but other times also very stressful.”* (Physician, 11 years of PC exp, male) P16

*“At the beginning I often have a certain tension, do I get into the conversation well?, do I find access to the person?”* (Nurse, 20 years of PC exp, female) P39

*“I am always anxious before any conversation, and I am very focused.”* (Nurse, 20 years of PC exp, female) P229

*“Calm concentration before the conversation so that I have everything important ready to share, and so that I am prepared for potential questions.”* (Physician, 17 years of PC exp, female) P30

During conversations, empathy and compassion were common, with initial relief through connection. A sense of clarity was experienced by most. If this was not achieved, a feeling of powerlessness or depression seemed to set in.

*“Before, usually also some nervousness/anxiety until I have found the “right thread” and I am in the topic with the client/relatives.”* (Nurse, 6 years of PC exp, female) P228

*“During the conversation, the entire situation relaxes or clears up, and at the end there is a sense of relief that the topic has been addressed and discussed. So, that a lot could be solved and clarified for the patient.”* (Nurse, 6 years of PC exp, female) P225

*“[During the conversation] most of the times serenity and empathy, sometimes frustration or sense of challenge (when the patient or relatives are totally inappropriate or in denial of the situation).”* (Physician, 7 years of PC exp, male) P8

*“In situations without open communication with affected individuals and relatives, I often feel depressed and helpless and powerless.”* (Nurse, 20 years of PC exp, female) P252

Post-conversation emotions depended on the course and outcome of the interaction: Successful conversations could lead to satisfaction, relief, and joy, and the feeling of having done something valuable and meaningful supported participants’ sense of well-being and satisfaction. However, ineffective conversations and the perception of not adding something positive to the situation often led participants to experience guilt, helplessness, uncertainty, or frustration.

*“Relief if a conversation has gone well. Disappointment and frustration when things don’t work out, especially when they don’t work out repeatedly. Sometimes uncertainty whether the contents addressed have been correctly understood.”* (Physician, 15 years of PC exp, female) P259

*“Emotions depend on whether the patient and relatives experienced me as a benefit in their situation or whether I was more of a nuisance and stirred things up too much.”* (Nurse, 6 years of PC exp, female) P96

Further, the experience of exhaustion and fatigue after conversations was common, mainly when emotions were intense.

*“After each conversation: some fatigue, need for a short island of time to recover.”* (Physician, 27 years of PC exp, male) P133

*“Emotional consternation and sympathy need a lot of strength and accordingly time to gain distance and to be able to continue with other professional tasks.”* (Nurse, 10 years of PC exp, female) P50

Clarifying the situation and aligning with patients’ views and wishes often energized clinicians, and having a plan or common goal, improved their comfort and motivation.

*“Shifting of the teams according to the new requirement gives a sense of shared competence and increased performance accountability.”* (Physician,15 years of PC exp, male) P4

*“Knowing that we are acting on behalf of those affected is extremely liberating for the work.”* (Nurse, 20 years of PC exp, female) P185

### Theme 2. Not always under control

Clinicians faced significant emotional challenges and felt overwhelmed, particularly when emotionally charged situations led to a perceived loss of control. We identified 3 subthemes encompassing the challenging situations that participants experienced: (1) confronted by clinical limitations and uncertainty, (2) challenging patient populations, topics, and complex family dynamics, and (3) interprofessional tensions.

#### Confronted by clinical limitations and uncertainty

Inability to control symptoms or burdening situations provoked helplessness and frustration, especially when patients were very emotional or when patients’ expectations were high. This sense of powerlessness intensified the emotional burden of their role.

*“Feeling of horror thinking about the still existing burden of symptoms despite the good drugs we have. Still great ignorance about standard pain treatment. […] Helplessness not to be able to change situations that are stressful.”* (Nurse, 16 years of PC exp, female) P67

Discussions about prognosis appeared to be more challenging for some and often included a feeling of uncertainty and fear of making mistakes when estimating life expectancy.

*“Uncertainty in conversations about prognosis.”* (Physician, <1 year of PC exp, male) P205

*“It is still difficult for me to call a family when I assess impending death. I’m afraid of making a mistake and giving a “false alarm.”* (Nurse, 4 years of PC exp, female) P65

#### Challenging patient populations, topics, and complex family dynamics

Young patients or families posed exceptional challenges, as their situation was often perceived as hard and unfair. Clinicians’ personal involvement often intensified based on patients’ age or family situation, complicating emotional management and professional boundaries.

*“If I am forced to take care of young people I do not feel well.”* (Nurse, 24 years of PC exp, female) P109

*“Patients and relatives who are closer to you or in a similar life situation, such as having children of the same age, are more difficult to manage. Emotionally, one is more connected. Setting boundaries is more difficult and can be stressful.”* (Nurse 12 years of PC exp, female) P61

*“Sometimes sadness and powerlessness when a fate is very hard, or the patients and relatives reacted very emotionally.”* (Physician, 15 years of PC exp, female) P139

Cultural differences and patients with cognitive impairment presented additional communication barriers. Denial of illness progression, unrealistic demands, or aggression toward the treating team also increased feelings of helplessness.

*“Communication with other cultures and religions remains difficult. Their needs are very different and not always compatible with our health care system and guidelines. A lot of mutual understanding and creativity is often required.”* (Nurse, 12 years of PC exp, female) P61

*“This [end of life] is a very difficult subject to discuss with patients in nursing homes, most of them have cognitive problems so they don’t feel concerned.”* (Nurse, 6 years of PC exp, male) P256

*“It is difficult when patients at the end of life still cling to “curative” treatments … difficult to address this.”* (Nurse, 8 years of PC exp, female) P113

*“Helplessness and despair when things went badly or when the relatives were aggressive or disrespectful with me or my colleagues. Even when the patient himself was calm and content.”* (Nurse, 25 years of PC exp, female) P231

Specific topics, like self-determined death, sexuality, and spirituality were topics individual participants had issues to address, often due to lack of training or preparedness.

*“I find the topic of self-determined dying a great challenge.”* (Nurse, 10 years of PC exp, female) P260

*“I can’t remember being uncomfortable during a conversation in recent years. However, I notice that I rarely bring up the subject of sexuality.”* (Nurse, 12 years of PC exp, female) P69

*“I always find the opening to the spiritual topic a bit bumpy. Under no circumstances do I want the patient to have the feeling that I want to convert him or her to something.”* (Physician, 5 years of PC exp, male) P151

#### Interprofessional tensions

In collaboration with professionals, anger arose when physicians external to the teams (e.g. oncologists or general practitioners) were not perceived as competent or sufficiently caring. Further, it was difficult when those treating physicians delayed information transmission and it was unclear to the nurse what to tell the patient. The quality of inter- and intraprofessional collaboration seemed clearer and less challenging within dedicated PC settings and when patients were fully under PC.

*“Horror at how patients are left alone by hospital doctors and oncologists.”* (Nurse, 16 years of PC exp, female) P67

*“Helpless or angry when I have to deal with family doctors who are not capable.”* (Nurse, 25 years of PC exp, female) P231

*“By working in the palliative setting, communication with patients and relatives became much easier, since much has already been decided or initiated. Previously, in the professional environment of a “curative” department with equally early palliative care patients or terminally ill people, communication was very difficult because many things were still unclear, could not be addressed, and the doctors/nursing team were not pulling in the same direction. So, I can say that in the appropriate environment, communication is much easier.”* (Nurse, 1 year of PC exp, female) P210

Misleading unclear information or action from other physicians, like not addressing the severity of the illness or the referral to a rehabilitation clinic when death was most likely imminent, put PC physicians in challenging situations with the dilemma of either destroying patients’ positivism or supporting false optimism.

*“I ask what exactly they know about their disease. Patients are often not aware of how advanced their condition is. I am sometimes annoyed with the referring physicians. After all, one doesn’t want to take away the patients’ enthusiasm. I sometimes feel like an accomplice to this false optimism.”* (Physician, 15 years of PC exp, male) P4

### Theme 3. Evolving comfort and competence through personal and professional growth

Despite the ongoing emotional challenges when communicating with patients in PC, clinicians generally reported improved comfort with EOL communication over their career. This growing comfort could be attributed to 6 different factors: (1) learning by doing and via formal training, (2) integrating life experiences and gaining insight from private life, (3) taking time, (4) collaborating and connecting with peers, (5) acknowledging individuality, and (6) connecting with one’s own and patients’ emotions.

#### Learning by doing and via formal training

Gaining experience and expertise over time enhanced comfort with communication. Successful interactions, positive and negative feedback from patients and supervisors, supported learning and improvement. Regarding content, the assimilation of strategies which supported successful conversation (e.g. patient centered communication or being authentic), the acquisition of appropriate formulations, as well as the accumulation of clinical experience and knowledge around treatment options and illness prognoses contributed to increased competence and security. Structured training complemented practical experience in developing skills and emotional resilience.

*“The experience brings a lot of security. You get the practical experience that you can “talk about anything.” I can’t remember being uncomfortable during a conversation in the last few years.”* (Nurse, 12 years of PC exp, female) P69

*“Through experience, it has generally become easier to address [palliative care] topics, since one has already found the right choice of words for oneself and knows how to start conversations.”* (Nurse, 2 years of PC exp, female) P59

*“Security increases with experience and positive feedback, but also with experience of why things are not going well.”* (Physician, 7 years of PC exp, female) P144

*“More experience makes it easier and easier. The more knowledge about diagnosis, prognosis, and treatment options, the easier the communication.”* (Nurse, 4 years of PC exp, female) P174

More specific knowledge through (further) education or training increased participants’ comfort and preparedness as they gained experience.

*“I was able to improve the quality of the discussions through the specialist knowledge acquired from further training, specialist forum, and specialist literature.”* (Nurse, 10 years of PC exp, female) P209

*“For me, communication courses, especially with simulations, were and are extremely helpful. You can practice and get direct feedback from the trainer and the “patient.”* (Physician, 12 years of PC exp, female) P6

Some participants therefore wanted further training. Beginners in PC wanted anamnesis guidance and other foundational skills. More experienced clinicians sought advanced communication training, particularly in discussing prognosis, therapy discontinuation, or working with children. Participants highlighted the need for structured training programs, role plays, and peer reviews to build proficiency and a confidence in these areas. There was also a recognition of their role in the bereavement phase, and therefore they also wanted training in grief counselling.

*“Due to the new activity in palliative care (2 months), there is still a relatively large uncertainty in many topics of conversation, which are not otherwise addressed in internal medicine. I would like to see structured training in communication skills to gain additional confidence in conducting such conversations (e.g. in the context of a training event, role plays, conversation process template in the sense of a “script,” alternatively: regular peer reviews).”* (Physician, <1 year of PC exp, male) P205

*“I would very much like to participate in a communication training on difficult topics such as prognosis, therapy discontinuation etc. Further training in grief counseling would also be enriching.”* (Nurse, 20 years of PC exp, female) P39

*“I would like to have more training around involving children in preparing for the approaching death of a parent.”* (Nurse, 8 years of PC exp, female) P201

While it was generally agreed that training was beneficial, some emphasized the need to gain experience by doing, which many felt could not be compensated with training or education.

*“Training is a resource, but experience remains fundamental.”* (Nurse, 21 years of PC exp, female) P38

Although not widespread, a participant held the perception that some aspects of communication and connection could not be taught because they were more like innate traits.

*“The quality of this communication and its modality are built throughout our career, our life path, our personal development, etc. Training is important in structuring this communication, but a significant part of it cannot be taught because it is an intrinsic part of us.”* (Physician, 10 years of PC exp, female) P121

#### Integrating life experiences and gaining insight from private life

Life experience had an impact on how participants coped with communication challenges. Participants saw their personal experiences as enriching their professional interactions and their professional identity. Many valued personal development and life experience as helpful.

*“The own development, and the own “aging” have helped a lot to experience these topics with more well-being during the conversations.”* (Physician, 12 years of PC exp, female) P5

Especially through the death of a loved one, clinicians felt that they could understand and use their experiences to connect with patients at a different level. The experience of having been “on the other side” as a patient or family caregiver also increased participants’ awareness of the importance of communication and of PC in general, leading them to be more compassionate and effective in their professional role with some even mentioning the benefits of self-disclosure during consultations.

*“Personal illness fates and the early loss of my parents (long accompaniment of one parent with a specialized palliative care team) have shown me that direct, appreciative communication is important and gave me courage to address things. The loss and mourning or also the accompaniment of relatives, have gained a completely new significance for me.”* (Nurse, 10 years of PC exp, female) P271

*“A severe personal loss in the family taught me what grieving means – since then I understand relatives much better.”* (Physician, 2 years of PC exp, female) P239

*“I encountered “Exit” through my father’s death wish. Since I see medically assisted suicide and palliative care as equivalent, many things have changed positively for me.”* (Physician 30 years of PC exp, female) P176

#### Taking time

For PC clinicians, *taking time* was important to prepare for the conversations, to be present during conversations, to create room during the conversations, and it helped process, recharge and focus on new tasks after the conversations. While some participants managed to have more control over how they used their time for communication, it depended the most on external factors, and therefore time pressure was still perceived as a common stressor.

*“I always take a lot of time when it comes to a palliative situation.”* (Physician, 15 years of PC exp, male) P235

*“Sometimes I get frustrated because I haven’t always been able to do what I would have liked to do due to lack of time.”* (Nurse, 18 years of PC exp, female) P56

*“In face-to-face conversations with people at the end of life, I think it’s important to listen, to be there, not to judge, and to bring time. Time is highly valued – and we must fight for this, both as nurses and as physicians, on one hand that we have enough time and on the other hand that this time is also compensated.”* (Nurse, 7 years of PC exp, female) P234

#### Collaboration and connecting with peers

Sharing unpleasant experiences with colleagues helped clinicians to process their emotions or solve issues, and even allowed some clinicians to handover tasks to colleagues when they felt overwhelmed. Physicians and nurses often described feeling secure within their teams to ask for this type of support in difficult cases. This collaborative approach was seen as key in providing good care for patients and for their own emotional relief.

*“I only get rid of the feeling of guilt through feedback of a colleague; I can’t do that myself.”* (Physician, 5 years of PC exp, male) P151

*“If I can or have to look after young people, I do not feel well. Then I need many conversations with my colleagues.”* (Nurse, 24 years of PC exp, female) P109

*“I have also experienced pure rejection, so that colleagues had to take over the entire care and support, and I withdrew.”* (Nurse, 20 years of PC exp, female) P39

*“[It helps that] I know where I can get information/help if I get stuck.”* (Nurse, 5 years of PC exp, female) P192

A connection with peers and experts through formal (e.g. Balint groups) or informal exchanges allowed participants to feel more secure and improve future interactions.

*“Constant dealing with these issues […] through peer exchange helps to develop calmness and security.”* (Physician, 12 years of PC exp, female) P6

*“Exchange with work colleagues, interprofessional case discussions [helped well-being].”* (Nurse, 9 years of PC exp, female) P223

Collaboration by involving peers and other professionals or even patients’ relatives seemed to improve participants’ comfort. This inclusive approach not only enriched the clinical decision-making process but also strengthened the support network around patients and their relatives.

*“I think for me it [the positive evolution] has to do with the experience and the well-trained interdisciplinary team that my employer offers. The exchange is very enriching. The cooperation with a hospice also has a positive effect.”* (Nurse, 10 years of PC exp, female) P218

*“[increased comfort because] I have become proactive in conversations, I get relatives more actively on board; I have more direct contact with family doctors/chaplains, etc. by phone and not just per mail.”* (Physician, 21 years of PC exp, female) P1

Working alone was described as more difficult. The absence of a team increased professional and personal challenges for the clinicians. This was more often the case for nurses working external to hospitals and providing services to patients at home. They often had little opportunities for connection or follow-up with colleagues or supervisors, which made them feel alone with the challenges they faced.

*“In home care, talking and intervening with patients and families alone is not easy. It would be much easier to have a team trained in palliative care and doing accompanied interventions.”* (Nurse, 20 years of PC exp, male) P41

#### Acknowledging and respecting individuality

The realization that there was not a right or wrong approach to each situation seemed to increase clinicians’ comfort. Understanding that patients’ wishes and priorities were individual and that conversations were not about the clinicians’ own values, was helpful. Focusing on listening and understanding without judgment, seemed to improve communication, comfort and sense of job fulfillment.

*“Good dying is defined by the individual, has many facets, my value system is relevant for me but not transferable to others, and is shaped by multicultural living environments, etc., is different every time.”* (Nurse, 15 years of PC exp, female) P40

*“In conversations with people at the end of life, I find it important to listen, to be there, not to judge.”* (Nurse, 7 years of PC exp, female) P234

*“Not everything always has to be discussed. Certain things can be addressed, but if someone does not want to talk about it, then just leave it. Sometimes it continues to work in the background and the topic gets taken up again later. Otherwise, it’s just okay the way it is.”* (Nurse, 6 years of PC exp, female) P225

#### Connecting with one’s own and patients’ emotions

Perceiving emotions consciously, allowing, and accepting them, including getting to know themselves and their own values increased comfort with communication challenges. Emotions were valued for they helped to connect and be empathic, and emotional awareness also allowed to detect internal resources and strengths (e.g. conveying calmness) and to realize personal boundaries.

*“Recognition, identification, and acceptance of my own emotions, allows me to be more serene with people.”* (Nurse, 19 years of PC exp, female) P45

*“I also personally did a psychoanalysis to get to know myself better what has been a very good investment.”* (Physician, 2 years of PC exp, female) P239

*“I try to transfer my inner peace to the patients and relatives.”* (Physician, 15 years of PC exp, male) P235

*“It is quite a wide range of feelings that can be perceived in such conversations. However, I find this important so that the conversation remains empathetic and authentic.”* (Nurse, 10 years of PC exp, female) P209

*“Today I listen much more to my gut.”* (Nurse, 12 years of PC exp, female) P254

However important the emotional experience was, participants still wanted balance, control, and distance, for their mental health and professional effectiveness.

*“I have never had a situation where I have been so overwhelmed that I was unable to address the topic, but I have always valued some “alone-time” afterwards to process what has just happened and its impact on me.”* (Nurse, 32 years of PC exp, male) P19

*“However, one’s own feelings must not be too strong, otherwise it becomes difficult to keep a clear head.”* (Nurse, 10 years of PC exp, female) P209

*“I have cried with a family after a death, I am often moved and share the sadness of the family but then I am able to get out of the situation and it doesn’t follow me home.”* (Physician, 29 years of PC exp, female) P94

Connecting with patients was seen as having a double-edged impact on own emotions: closer relationships led to increased sadness, less perceived professionalism, and difficulties keeping mental distance from work, but also seemed to improve clinician well-being and to help patients open up.

*“Easier [to communicate] if I have already built up a certain nursing relationship and in the course of everyday nursing already pick up wishes or statements and incorporate them into the conversation, also the relationship makes such a conversation more familiar and open.”* (Nurse, 3 years of PC exp, female) P103

*“Peace, if I already knew the people before the conversation. Grief, if I knew the people personally.”* (Nurse, 25 years of PC exp, female) P231

*“With families and loved ones, I often feel like a worn-out washcloth. Professionalism is no longer perceptible to me.”* (Nurse, 24 years of PC exp, female) P262

Overall, most participants were self-aware and could identify their emotions and the situations that led to them. Only 1 participant stated difficulties identifying her emotions during conversation, and another participant questioned her capacity to remain self-aware and whether PC was a job that she could do for long.

*“Self-care or self-awareness definitely comes up short for me. I do not know how long I can do this work.”* (Nurse, 8 years of PC exp, female) P37

*“During [the conversation] no idea [what the emotions are].”* (Nurse, 4 years of PC exp, female) P65

Over time, most participants not only recognized their own emotions, but also improved the verbalization of their and their counterparts’ emotions, which seemed to support emotion processing, and more open communication.

*“The experience has allowed me to be comfortable talking about my own feelings and those of the patient and their loved ones.”* (Physician, 10 years of PC exp, female) P121

*“Even if certain topics are difficult to discuss, such as talking about one’s own feelings or how one is affected, I have learned that this is also a valuable and important part.”* (Nurse, 6 years of PC exp, female) P225

Participants also recognized patient emotions and needs, which increased respect and understanding for patients, but also made it possible to notice their relief and gratitude. The feeling of having contributed to patients’ well-being made professionals feel rewarded and the feeling of doing something meaningful contributed to feeling more comfortable.

*“[It got easier] to acknowledge and accept the emotions of the patient and his or her loved ones, even if it is sometimes a little more difficult with the relatives.”* (Nurse, 7 years of PC exp, female) P253

*“Greater sensitivity to the needs of the patient. Better able to put aside one’s own opinion.”* (Nurse, 10 years of PC exp female) P248

*“Feeling of being supportive in the process, a confidant, a person who gives support and strength, of being a competent contact person in terms of care.”* (Nurse, 15 years of PC exp, male) P279

*“The relatives live on with the images of death, therefore the situation should be coordinated as well as possible. With my professional experience, I usually succeed communicating with them, and this shows me the meaning of my work.”* (Nurse, 10 years of PC exp, female) P222

Finally, in addition to being aware of emotions, facing and processing them seemed to help. The majority of strategies, most already mentioned, such as expressing emotions, evaluating the conversations, or reflecting on own feelings, etc. reinforced the existence of emotions rather than denied them, for example by using strategies such as seeking distraction, which were not reported by any of the participants.

## Discussion

This study explored PC clinicians’ experiences of EOL communication and the evolution of their comfort and skills throughout their career. Our findings highlight the inherent emotional complexity of PC communication, involving a range of emotions that can both challenge and enrich clinical care. This emotional complexity underscores the significant emotional labor involved in PC communication, which requires ongoing management and adaptation.

The experience of burdening emotions and anxiety reported by participants, align with existing research on the risk of burnout in EOL care and why the emotional side of communication needs to be acknowledged more widely (Horn and Johnston 2020; Koh et al. 2015; Parola et al. 2017; Pereira et al. 2011). Factors influencing negative emotional experiences included lack of knowledge or experience, difficulties in collaboration with patients or colleagues, or working with young patients. These findings align with previous studies which have identified: uncertainty about prognosis, feeling unprepared, and young patient age as barriers to open communication, while teamwork and colleagues have been highlighted as a valuable source to manage difficult EOL situations (Brighton and Bristowe 2016; Brighton et al. 2019; Fulmer et al. 2018; Gonella et al. 2023; Mack et al. 2021; You et al. 2015).

Alongside challenges, participants experienced substantial rewards including joy, satisfaction, and feelings of privilege in accompanying patients at the EOL. This balance between stress and reward of PC work has been previously described, including their need to find a balance (Kearney et al. 2009; Lehto et al. 2020; Zambrano et al. 2012, 2014). In our study, factors contributing to that balance included personal and professional experience, training, time availability, recognizing the patients’ individuality, emotional engagement, and collegial support. These elements highlight the importance of a wide-encompassing approach to clinician support, focusing not only on skills training but also on increasing the availability and access to emotional and relational support mechanisms. Our findings echo the need for developing emotional competence as a core professional skill, including self-awareness, and emotional regulation. Awareness of own and others’ emotions facilitated accepting and valuing the views and priorities of others and seemed to give clinicians more room to express and share own emotions. This awareness is believed to be a key variable to clinicians’ quality of life (Galiana et al. 2015), work satisfaction, and to quality of patient care (Novack et al. 1997), as unrecognized feelings can hinder empathy or meaningful discussions and may lead to under or overinvolvement with certain patients (Novack et al. 1997). To enhance personal awareness and mindfulness different approaches exist (Novack et al. 1997), including practices highlighted by our participants and supported by literature, such as considering the audience, asking questions, listening, and recognizing one’s role (Omilion-Hodges and Swords 2016), finding out the counterparts’ perspective first, or displaying sensitivity (Ekberg et al. 2021).

A key finding was also the interplay between personal and professional identities in shaping communication strategies. Participants’ actively integrated personal experiences as patients or caregivers into their professional practice, enriching their communication and connection with patients. This integration aligns with studies showing how personal loss can contribute to compassionate presence and professional development (Supiano and Vaughn-Cole 2011), suggesting that personal life experiences and self-disclosure can be integrated thoughtfully within professional practice to enhance empathy and effective communication. A balance between integrating personal experience and respecting the patient’s individuality can also enhance shared-decision making processes.

### Clinical implications

Despite advances in communication training, our findings suggest that expertise in PC communication largely develops outside formal training structures and is fraught with challenges that could be mitigated through earlier and more comprehensive support.

Integrating emotional competence training and reflective practices into core curricula (Schwartz et al. 2021), rather than treating them as auxiliary skills that develop with experience, could better prepare clinicians for the emotional demands of PC. Furthermore, fostering a culture that acknowledges and values the role of emotions in health care from the outset of professional education could help alleviate the sense of isolation and self-doubt many clinicians experience when navigating complex emotional dynamics early in their careers (Slavin 2021). Reframing emotions as informative rather than disruptive can inform clinicians’ approach to patient care, rather than seeing them as personal weaknesses (Childers et al. 2021). Training programs should thus focus on building emotional resilience, integrating emotional well-being, self-awareness, and self-care practices (Galiana et al. 2015) as essential in preparing clinicians for the multifaceted challenges of PC communication. Furthermore, training programs should not only provide foundational skills but also offer continuous professional development opportunities tailored to the evolving needs of clinicians. This includes addressing specific areas such as cross-cultural EOL communication (Cain et al. 2018; Semlali et al. 2020), *cultural humility* (Neubauer et al. 2015), and specific challenging topics like spirituality, prognosis, self-determined/hastened death, or working with extra-challenging patient groups, such as children. Reducing these stressors and barriers, particularly for the younger clinicians and those having difficulties in initiating these conversations could support early discussions and help improve patient care. Finally, minimizing barriers to EOL conversations through adequate time allocation, supporting collaboration among colleagues, open communication, and teamwork, and including structures that allow for reflective practice and peer support are among the required organizational changes that can significantly enhance the quality and effectiveness of PC communication, ultimately leading to improved patient care and clinician and team well-being (Omilion-Hodges et al. 2020).

### Strengths and limitations

A potential limitation of the study is that unlike with in-depth interviews, the survey approach prevented us from posing follow up questions to participants. Moreover, qualitative surveys typically gain breadth rather than depth capturing a wide range of experiences but potentially missing nuanced details (Terry and Braun 2017), however, our large sample of 231 clinicians from different institutions provides meaningful insights into experiences of EOL communication. Hearing a wide range of voices is known to be especially useful in sensitive areas and the felt anonymity could have led to more open responses, particularly when discussing emotions in medicine (Braun et al. 2021). Further, recall bias might have led to discrepancies between what was actually felt and how participants remembered and evaluated their prior experiences (Colombo et al. 2020). Finally, we acknowledge the role of our subjectivity in the interpretation of the results, however in line with reflexive thematic analysis, we view it as resource rather than a limitation.

## Conclusion

To effectively navigate the emotional complexity of PC communication, clinicians require engagement and emotional competence as core professional skills, which continually evolve with training and experience. Our study underscores the importance of integrating personal and professional experiences in developing emotional resilience and effective communication strategies in PC. Educational, structural, and organizational improvements are necessary to support these competencies. By integrating these insights into education and practice, we can further enhance clinicians’ ability to provide compassionate, patient-centered care in the face of the profound emotional challenges inherent in PC practice.
